# Type of arteriovenous vascular access and association with patency and mortality

**DOI:** 10.1186/1471-2369-14-79

**Published:** 2013-04-04

**Authors:** Gürbey Ocak, Joris I Rotmans, Carla Y Vossen, Frits R Rosendaal, Raymond T Krediet, Elisabeth W Boeschoten, Friedo W Dekker, Marion Verduijn

**Affiliations:** 1 Department of Clinical Epidemiology, Leiden University Medical Center, Leiden, The Netherlands; 2 Department of Nephrology, Leiden University Medical Center, Leiden, The Netherlands; 3 Division of Biomedical Genetics, Department of Medical Genetics, University Medical Center Utrecht, Utrecht, The Netherlands; 4 Department of Thrombosis and Haemostasis, Leiden University Medical Center, Leiden, The Netherlands; 5 Department of Nephrology, Academic Medical Center, Amsterdam, The Netherlands; 6 Hans Mak Institute, Naarden, The Netherlands

**Keywords:** Hemodialysis, Fistula, Graft, Patency, Mortality, Epidemiology

## Abstract

**Background:**

There are only a few risk factors known for primary patency loss in patients with an arteriovenous graft or fistula. Furthermore, a limited number of studies have investigated the association between arteriovenous access modality and primary patency loss and mortality. The aim of this study was to investigate risk factors for patency loss and to investigate the association between graft versus fistula use and outcomes (patency loss and mortality).

**Methods:**

We prospectively followed 919 incident hemodialysis patients and calculated hazard ratios (HRs) for putative risk factors of primary patency loss using Cox regression. Furthermore, HRs were calculated to study the association between graft versus fistula use and two-year primary patency loss and two-year mortality.

**Results:**

Cardiovascular disease, prior catheter use, lowest tertile of albumin, highest tertile of hsCRP, and lowest tertile of fetuin-A were associated with primary patency loss in both patients with grafts and fistulas. Increased age, female sex, and diabetes mellitus were only associated with primary patency loss in patients with a fistula. We did not observe an association between primary patency loss and BMI, residual GFR, levels of calcium, phosphorus, and total cholesterol. Furthermore, graft use as compared with fistula use was associated with an 1.4-fold (95% CI 1.0-1.9) increased risk of primary patency loss and with an 1.5-fold(95% CI 1.0-2.2) increased mortality risk.

**Conclusion:**

Cardiovascular disease, prior catheter use, albumin, hsCRP, and fetuin-A are risk factors for patency loss. Graft use as compared with fistula use was associated with an increased risk of patency loss and mortality.

## Background

Preservation of adequate vascular access is of vital importance for patients undergoing chronic hemodialysis. Vascular access-related morbidity accounts for 20% of all hospitalizations in hemodialysis patients leading to high costs [1,2].

Several studies have shown that graft use as compared with fistula use was associated with an increased risk of patency loss [3]. However, few studies have investigated whether risk factors for patency loss are different for fistula use and graft use. The vast majority of arteriovenous access failure is caused by thrombosis, secondary to disproportionate intimal hyperplasia in the venous outflow tract [4-7]. The mechanisms that are responsible for this localized hyperplastic response are incompletely understood. The prevailing opinion is that local hemodynamic factors such as flow turbulence, vascular inflammation as well as the prothrombotic milieu that results from endothelial damage play a role in the formation of stenotic lesions [8,9]. Factors associated with atherosclerotic vascular disease and inflammation might play a different role in the formation of stenotic lesions in fistulas and graft. CRP and fetuin-A are both markers for inflammation and cardiovascular disease that could be associated with patency loss.

Moreover, a limited number of studies have investigated the association between fistula use versus graft use and mortality [10,11]. These studies found a moderately increased mortality risk for graft use as compared with fistula use [10,11]. The National Kidney Foundation Kidney Disease Outcome Quality Initiative guidelines [12] and the European Best Practice Guidelines [13] recommend the use of a fistula instead of grafts for vascular access in all hemodialysis patients. To date, it is unknown whether the graft use versus fistula use is associated with both patency loss and mortality.

In the present study, we investigated risk factors for primary patency loss (i.e. any intervention in the arteriovenous access after the first successful cannulation) in a large Dutch cohort of 919 incident hemodialysis patients [14]. In addition, we investigated the association between graft use versus fistula use and two-year patency loss and mortality.

## Methods

### Patients

The Netherlands Cooperative Study on the Adequacy of Dialysis (NECOSAD) is a prospective multicenter cohort study in which incident adult end-stage renal disease patients from 38 dialysis centers in the Netherlands were included. The study was approved by all local medical ethics committees (Maasstad Hospital Rotterdam, Deventer Hospital Deventer, Sint Lucas Andreas Hospital Amsterdam, Dianet Dialysis Center Academic Medical Center Amsterdam, Maxima Medical Center Veldhoven, Catharina Hospital Eindhoven, Medical Center Haaglanden Den Haag, University Medical Center Groningen, Kennemer Gasthuis Haarlem, Atrium Medical Center Heerlen, Medical Center Leeuwarden, Leiden University Medical Center Leiden, Elisabeth Hospital Tilburg, University Medical Center Utrecht, Antonius Ziekenhuis Nieuwegein, Hospital Gelderse Vallei Ede, Haga Hospital Leyenburg Den Haag, Academic Hospital Maastricht, Jeroen Bosch Hospital Den Bosch, Medisch Spectrum Twente Enschede, Albert Schweitzer Hospital Dordrecht, Alysis Zorggroep Rijnstate Hospital Arnhem, Dianet Dialysis Center Lunetten Utrecht, Canisius Wilhelmina Hospital Nijmegen, Vie Curi Medical Center Venlo, Leveste Scheper Hospital Emmen, Dianet Dialysis Center Holendrecht Amsterdam, Hage Hospital Rode Kruis Den Haag, Rijnland Hospital Leiderdorp, Admiraal de Ruyter ziekenhuis Goes, Medical Center Alkmaar, Laurentius Ziekenhuis Roermond, Dialysis Center ’t Gooi Hilversum, Groene Hart Hospital Gouda, Westfries Gasthuis Hoorn, Tergooiziekenhuizen Hospital Hilversum, Martini Ziekenhuis Groningen, Zaans Medical Center Zaandam). We followed patients until death or censoring, i.e. transfer to a nonparticipating dialysis center, withdrawal from the study, transplantation, or end of the follow-up period (April 2006). We did not censor for patency loss when investigating the effect of fistula versus graft use on mortality.

Eligibility included age older than 18 years, no previous renal replacement therapy, and survival of the initial three months of dialysis. For the current analyses, we used data from incident hemodialysis patients included between January 1997 and April 2004 with a functional arteriovenous access (native fistulas or grafts) within three months after the first dialysis session. Information about graft use or fistula use was collected from the medical records of patients. All patients gave informed consent.

### Demographic and clinical data

Data on age, sex, body mass index (BMI), primary kidney disease, cardiovascular disease (angina pectoris, myocardial infarction, heart failure, ischemic stroke, or claudication), and diabetes mellitus were collected at the start of dialysis treatment. BMI was calculated as weight in kilograms divided by height in meters squared. Primary kidney disease was classified according to the codes of the European Renal Association-European Dialysis and Transplant Association (ERA-EDTA) [15]. We grouped patients into four classes of primary kidney disease: glomerulonephritis, diabetes mellitus, renal vascular disease, and other kidney diseases. Other kidney diseases consisted of patients with interstitial nephritis, polycystic kidney diseases, other multisystem diseases, and unknown diseases.

The following biochemical parameters were routinely measured in blood samples obtained from patients at 3 months after the start of dialysis: creatinine, urea, calcium, phosphorus, albumin, total cholesterol. Renal function, expressed as glomerular filtration rate (GFR), was calculated as the mean of creatinine and urea clearance corrected for body surface area (ml/min per 1.73 m^2^). Moreover, circulating fetuin-A serum levels and high-sensitivity C-reactive protein (hsCRP) were measured at 3 months after the start of dialysis as described elsewhere [12].

### Primary patency loss and mortality

Time to primary patency loss was defined as the interval from time of first successful cannulation of the vascular access for hemodialysis treatment (first dialysis session) to surgery, percutaneous endovascular intervention, or abandonment of the vascular access in the first two years on dialysis. Information about surgery, percutaneous endovascular intervention, or abandonment of the vascular access was obtained from the standard data collection of NECOSAD. Two-year mortality was recorded according to the codes of the ERA-EDTA [15].

### Statistical analysis

Continuous variables are presented as median and interquartile range (IQR). Categorical variables are presented as number with percentages. We calculated hazard ratios (HRs) with 95% confidence intervals (95% CIs) using Cox regression analysis. HRs were calculated for primary patency loss within two-years of follow-up for previous catheter use, factors associated with atherosclerotic vascular disease (age, sex, diabetes mellitus, body mass index, residual glomerular filtration rate (GFR), calcium levels corrected for albumin, phosphorus levels, cholesterol levels, and fetuin-A levels), and factors associated with inflammation (hsCRP and albumin) stratified for patients with a fistula and patients with a graft use. Confounders were defined as variables that could be associated with exposure and with outcome, based on previous literature, without being an intermediate variable in the causal pathway between exposure and outcome [13]. Therefore, each investigated variable could have a different set of variables that were adjusted for. For the same reason, the effect of sex was not adjusted for other variables.

Survival curves for primary patency loss and mortality were determined with the Kaplan–Meier method stratified for fistula use and graft use. We calculated crude and adjusted hazard ratios (HRs) with 95% confidence intervals (95% CIs) for primary patency loss and mortality within 2 years of follow-up for patients with a fistula and patients with a graft using Cox regression analysis. All analyses have been done in SPSS statistical software version 18.0 (SPSS, Chicago, Ill).

## Results

A total of 919 incident hemodialysis patients with an arteriovenous access were included in NECOSAD. Of the 919 patients, 727 had a fistula, 146 had a graft, and 46 patients had an unknown type of arteriovenous access. Patients with a graft were older, were more often female, had a higher body mass index, had more often diabetes mellitus as primary kidney disease, had lower residual GFR and had higher total cholesterol levels than patients with a fistula (P<0.05) (Table 1).

**Table 1:** Baseline characteristics of patients with a fistula and graft

	**Fistula**		**Graft**	
	**N=727**		**N=146**	
Age (years) (IQR)	65.8	(54.5-73.7)	68.5	(59.4-74.3)
Sex, female (%)	37.4		59.6	
BMI (kg/m^2^) (IQR)	24.0	(22.0-26.7)	24.8	(21.5-27.8)
Primary kidney disease (%)				
Diabetes mellitus	14.7		17.8	
Glomerulonephritis	12.0		5.5	
Renal vascular disease	19.1		28.1	
Others	54.2		48.6	
Cardiovascular disease (%)	40.0		42.5	
Prior catheter use (%)	26.8		32.2	
Systolic blood pressure, mmHg	148	(135–162)	144	(133–158)
GFR (ml/min/1.73 m^2^) (IQR)	3.2	(1.7-5.0)	2.4	(1.1-4.4)
Calcium (mmol/L) (IQR)	2.4	(2.2-2.5)	2.4	(2.2-2.6)
Phosphorus (mmol/L) (IQR)	1.8	(1.5-2.2)	1.8	(1.5-2.2)
Serum cholesterol (mmol/L) (IQR)	4.6	(3.9-5.4)	4.9	(4.1-6.0)
Serum albumin (g/L) (IQR)	37	(34–40)	36	(32–40)
hsCRP* (mg/L) (IQR)	5.3	(2.0-13.8)	6.5	(2.5-16.7)
Fetuin-A* (g/L) (IQR)	0.6	(0.5-0.7)	0.6	(0.5-0.8)

BMI, body mas index; IQR, interquartile range.

*Missing in 276 patients with a fistula and 63 patients with a graft.

**Figure 1:**
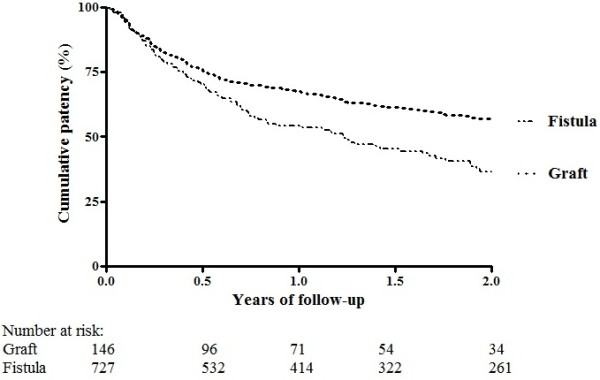
Kaplan-Meier survival curve for two-year primary patency loss after first successful cannulation.

**Figure 2:**
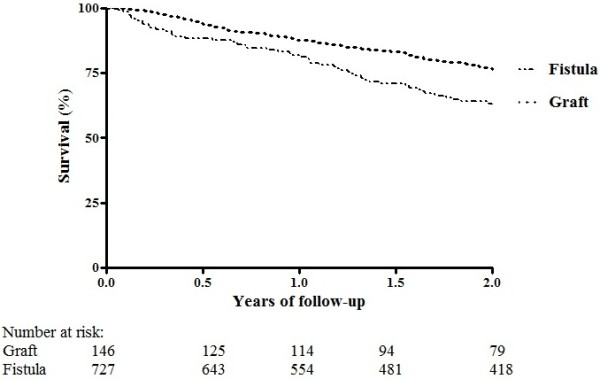
Kaplan-Meier survival curve for two-year mortality.

During the two-year follow-up, 287 of the 727 patients with a fistula and 84 of the 146 patients with a graft lost primary patency of their vascular access within two years. The cumulative incidence at two years for primary patency at two years was 56.8% for patients with a fistula and was 36.4% for patients with a graft (Figure 1). Of the 727 patients with a fistula, 149 died within 2 years. Of the 146 patients with a graft, 51 died within 2 years. Figure 2 shows the Kaplan-Meier survival curve with two-years mortality as outcome for patients with a fistula and graft. The cumulative survival was 76.4% for patients with a fistula and 63.2% for patients with a graft.

Table 2 shows the adjusted hazard ratios of the risk factors for primary patency loss stratified for patients with a fistula and patients with graft. Increased age ≥65 years (HR 1.3; 95% CI 1.0-1.7), female sex (HR 1.5; 95% CI 1.2-2.9), and diabetes mellitus (HR 2.0; 1.4-2.7) were associated with an increased risk of primary patency loss for patients with a fistula and not for patients with a graft. Cardiovascular disease (HR 1.7; 95% CI 1.3-2.2 and HR 1.8; 95% CI 1.1-2.9), prior catheter use (HR 1.9; 95% CI 1.5-2.4 and HR 2.1; 95% CI 1.3-3.4), lowest tertile of albumin (HR 1.5; 95% CI 1.1-2.1 and HR 2.4; 95% CI 1.3-4.5), highest tertile of hsCRP (HR 1.6; 95% CI 1.1-2.3 and HR 2.7; 95% CI 1.2-6.3), and lowest tertile of fetuin-A (HR 1.9; 95% CI 1.3-2.9 and HR 3.6; 95% CI 1.7-7.4) were associated with primary patency loss after adjustment in both patients with a fistula and graft, respectively. We did not find an association between primary patency loss and BMI, GFR, levels of calcium corrected for albumin, phosphorus, and cholesterol after adjustment (Table 2).

**Table 2:** Risk factors for primary patency loss

			**Fistula**				**Graft**			
			**N=727 hazard ratio (95% CI)**				**N=146 hazard ratio (95% CI)**			
			**Crude**		**Adjusted**		**Crude**		**Adjusted**	
Age* (years)	≥65 versus <65	1.3		(1.1-1.7)	1.3	(1.0-1.7)	1.1	(0.7-1.7)	1.1	(0.7-1.7)
Sex^†^	Female versus Male		1.5	(1.2-2.9)	1.5	(1.2-1.9)	0.7	(0.5-1.1)	0.7	(0.5-1.1)
BMI^‡^ (kg/m^2^)	≥25 versus <25		1.0	(0.8-1.2)	0.9	(0.7-1.1)	0.8	(0.5-1.3)	0.8	(0.5-1.3)
Primary kidney disease^§^	Diabetes mellitus		2.1	(1.6-2.9)	2.0	(1.4-2.7)	1.4	(0.8-2.5)	1.2	(0.7-2.1)
	Glomerulonephritis		0.8	(0.6-1.3)	0.9	(0.6-1.3)	1.1	(0.4-3.1)	1.2	(0.4-3.5)
	Vascular disease		1.0	(0.8-1.4)	0.9	(0.7-1.3)	0.9	(0.5-1.5)	0.7	(0.4-1.2)
	Others		1	(reference)	1	(reference)	1	(reference)	1	(reference)
Cardiovascular disease^¶^	Yes versus No		1.7	(1.4-2.2)	1.7	(1.3-2.2)	1.8	(1.2-2.8)	1.8	(1.1-2.9)
Prior catheter use**	Yes versus No		1.9	(1.5-2.4)	1.9	(1.5-2.4)	2.2	(1.4-3.4)	2.1	(1.3-3.4)
Systolic blood pressure**	Low	<139	0.9	(0.7-1.2)	0.9	(0.7-1.2)	1.7	(1.0-2.9)	1.5	(0.8-2.6)
(mmHg)	Median	139-155	1.0	(0.8-1.4)	1.1	(0.8-1.4)	1.0	(0.6-1.8)	1.1	(0.6-2.0)
	High	>155	1	(reference)	1	(reference)	1	(reference)	1	(reference)
GFR**	>10		1	(reference)	1	(reference)	1	(reference)	1	(reference)
(ml/min/1.73 m^2^)	5-10		1.1	(0.5-2.3)	1.0	(0.5-2.1)	0.5	(0.1-1.6)	0.6	(0.2-2.1)
	<5		0.9	(0.5-1.9)	0.9	(0.4-1.7)	0.4	(0.1-1.3)	0.4	(0.1-1.4)
Calcium** (mmol/L),	Low	<2.30	1	(reference)	1	(reference)	1	(reference)	1	(reference)
Tertiles	Median	2.30-2.49	1.3	(0.9-1.7)	1.3	(1.0-1.7)	1.0	(0.6-1.8)	1.1	(0.6-1.9)
	High	>2.49	1.0	(0.8-1.4)	1.0	(0.8-1.4)	1.4	(0.8-2.3)	1.5	(0.9-2.5)
Phosphorus** (mmol/L),	Low	<1.57	1	(reference)	1	(reference)	1	(reference)	1	(reference)
Tertiles	Median	1.57-2.02	0.9	(0.7-1.2)	0.9	(0.7-1.2)	0.7	(0.4-1.2)	0.8	(0.4-1.3)
	High	>2.02	0.9	(0.7-1.2)	0.9	(0.7-1.2)	0.9	(0.5-1.5)	0.9	(0.5-1.5)
Cholesterol** (mmol/L),	Low	<4.20	1	(reference)	1	reference)	1	(reference)	1	(reference)
Tertiles	Median	4.20-5.10	0.7	(0.5-0.9)	0.7	(0.5-1.0)	0.9	(0.5-1.6)	0.9	(0.5-1.6)
	High	>5.10	0.8	(0.6-1.1)	0.8	(0.6-1.1)	0.7	(0.4-1.2)	1.0	(0.5-1.7)
Albumin** (g/L),	Low	<35.0	1.8	(1.3-2.4)	1.5	(1.1-2.1)	3.1	(1.7-5.5)	2.4	(1.3-4.5)
Tertiles	Median	35.0-38.9	1.3	(1.0-1.8)	1.2	(0.9-1.7)	2.1	(1.1-4.0)	2.4	(1.2-4.6)
	High	>38.9	1	(reference)	1	(reference)	1	(reference)	1	(reference)
hsCRP** (mg/L),	Low	<2.95	1	(reference)	1	(reference)	1	(reference)	1	(reference)
Tertiles	Median	2.95-9.94	1.1	(0.7-1.6)	1.1	(0.7-1.6)	2.5	(1.1-5.5)	2.6	(1.0-6.5)
	High	>9.94	1.6	(1.1-2.4)	1.6	(1.1-2.3)	2.5	(1.1-5.6)	2.7	(1.2-6.3)
Fetuin-A** (g/L), Tertiles	Low	<0.55	1.9	(1.3-2.9)	1.9	(1.3-2.9)	3.6	(1.4-6.7)	3.6	(1.7-7.4)
	Median	0.55-0.64	1.5	(1.0-2.3)	1.5	(1.0-2.3)	3.1	(1.8-7.3)	3.3	(1.5-7.5)
	High	>0.64	1	(reference)	1	(reference)	1	(reference)	1	(reference)

*Adjusted for sex, ^†^Unadjusted, ^‡^Adjusted for age, sex, primary kidney disease, and cardiovascular disease, ^§^Adjusted for age, sex, BMI, and cardiovascular disease, ^¶^Adjusted for age, sex, BMI, and primary kidney disease, **Adjusted for age, sex, BMI, primary kidney disease, and cardiovascular disease.

Graft use as compared with fistula use was associated with an 1.4-fold (95% CI 1.0-1.9) increased risk of primary patency loss and with an 1.5-fold (95% CI 1.0-2.2) increased two-year mortality risk after adjustment for age, sex, BMI, primary kidney disease, cardiovascular disease, prior catheter use, and levels of calcium, phosphorus, and cholesterol (Table 3).

**Table 3:** Association between graft versus fistula and patency loss and mortality

	**Type of access**			**Patency loss**		**Mortality**	
				**HR (95% CI)**		**HR (95% CI)**	
**Total**	Fistula	(N=727)		1	(reference)	1	(reference)
	Graft	(N=146)	Crude	1.6	(1.3-2.0)	1.7	(1.3-2.4)
			Adjusted*	1.4	(0.9 -2.1)	1.5	(1.0-2.2)

HR, hazard ratio; CI, confidence interval.

*Adjusted for age, sex, BMI, primary kidney disease, cardiovascular disease, prior catheter use, GFR, calcium corrected for albumin, phosphorus, and cholesterol.

## Discussion

In this prospective cohort study of 919 incident hemodialysis patients with an arteriovenous access, we showed that cardiovascular disease, prior catheter use, levels of albumin, hsCRP, and fetuin-A were associated with primary patency loss in both patients with a fistula and a graft. Increased age, female sex, and diabetes mellitus was only associated with an increased risk of primary patency loss in patients with a fistula. We did not find an association between primary patency loss and BMI, GFR, and levels of calcium, phosphorus, and cholesterol. Furthermore, we showed that graft use as compared with fistula use was associated with an 1.4-fold (95% CI 1.0-1.9) increased risk of primary patency loss and with an 1.5-fold (95% CI 1.0-2.2) increased two-year mortality risk.

Previous smaller studies on risk factors of arteriovenous dysfunction have shown conflicting results [16-23]. Increased age has been associated in previous studies with vascular access morbidity [16,17,19], but not in other studies [18,20,21]. Similar inconsistencies have been observed in previous studies on gender as a risk factor for arteriovenous access dysfunction [18-21,23]. In our large study using incident hemodialysis patients with an arteriovenous access, we have showed that increased age, female gender, and diabetes mellitus were associated with primary patency loss in patients with a fistula and not in patients with a graft. Since patients with a graft are a group of selected dialysis patients with an increased mortality risk and an increased risk of patency loss as compared with patients with a fistula, selection bias could explain the differences in the association between patency loss and age, sex, and presence of diabetes mellitus. Another reason could be that graft patency is less influenced by age, sex, and diabetes mellitus than fistula patency. Furthermore, we had less power in the graft group than in the fistula group for the investigation of risk factors for patency loss. In line with our study, cardiovascular disease [16,19] and prior catheter use [22] have been shown to be important risk factors for arteriovenous access dysfunction in other studies.

Limited studies have investigated the association between arteriovenous access dysfunction and BMI, GFR, calcium, phosphorus, and cholesterol. In concordance with the results of previous studies [17-19], these potential risk factors were not associated with arteriovenous access dysfunction in our study. Fetuin-A, hsCRP, and albumin levels have been associated with mortality in dialysis patients [23-26]. However, a new observation in our study was that levels of fetuin-A, hsCRP, and albumin levels were associated with vascular access dysfunction in patients with a fistula and in patients with a graft.

The pathogenic mechanisms for arteriovenous dysfunction are incompletely understood, but it is thought that thrombosis resulting from stenosis due to neointimal hyperplasia is the main cause of arteriovenous dysfunction [7-9]. The stimuli responsible for this localized intimal hyperplastic response in the venous outflow tract are multifactorial and include hemodynamic factors such as turbulent flow, endothelial damage as well as repetitive strain injury and vascular inflammation that might relate to compliance mismatch between the anastomosed blood vessels [7-9]. The stenotic vascular lesions that arise from this intimal hyperplastic response mainly consist of vascular smooth muscle cells, myofibroblasts and extracellular matrix proteins such as collagen [7-9]. A recent study showed that the stenotic vascular lesions are already present prior to dialysis access placement [27]. Morphologically, these lesions differ substantially from atherosclerotic lesions that mainly consist of lipid-rich foam cells and activated T-cells [28,29]. Interestingly, our study suggests that well-known risk factors for atherosclerosis (cardiovascular disease and fetuin-A levels) and factors associated with inflammation (C-reactive protein and albumin) might play an important role in the development of stenotic lesions in arteriovenous fistulas in dialysis patients as well.

In the present study, graft use as compared with fistula use was associated with an 1.4-fold (95% CI 1.0-1.9) increased risk of primary patency loss and with an 1.5-fold (95% CI 1.0-2.2) increased two-year mortality risk. Previous studies found also an increased risk of patency los in patients with a graft as compared with patients with a fistula. However, limited studies have investigated the association between type of arteriovenous access and mortality [10,11]. These studies suggested an increased mortality risk for graft use as compared with fistula use [10,11]. Although National Kidney Foundation Kidney Disease Outcome Quality Initiative guidelines [12] and the European Best Practice Guidelines [13] recommend the use of a fistulas instead of grafts for vascular access in all hemodialysis patients, it could be that for special subgroups, such as elderly patients, grafts are good alternatives as first option for a vascular access, especially when we take into account that failure of vascular access before successful cannulation for dialysis is higher for fistulas than for grafts [30].

Our study has several potential limitations. We had no information on several vascular access characteristics (anatomic location, flow, vessel diameter, and intervention prior to cannulation) that are associated with vascular access dysfunction. Moreover, type of arteriovenous access was unknown in 46 patients. However, when unknown type of vascular access was either classified as graft use or fistula, the influence of these unknown type of vascular accesses in the association between graft use versus fistula use and patency loss or mortality was minimal (data not shown). Another limitation is that confounding-by-indication could occur when comparing different outcomes for graft use versus fistula use in an observational design. The observed increased mortality risk and patency loss of graft use versus fistula use may partly reflect the effect of other differences between graft and fistula users. In our analyses, we took this into account by correcting for many confounders, but this cannot exclude possible residual confounding. Therefore, randomized controlled trials are needed when comparing outcomes between graft use and fistula use. However, there might be ethical and practical problems to conduct this kind of a randomized controlled trial. In view of the clinical importance in combination with the small differences in outcomes between graft use and fistula use in elderly patients, ethical objections against such a randomized controlled trial seem exaggerated. Furthermore, we had no information about failure of arteriovenous accesses before the successful first cannulation. However, this would probably result in an underestimation of the point estimates for the investigated risk factors. The general strength of this study was the large and well-defined Dutch cohort of incident hemodialysis patients with an arteriovenous access with available data on many patient characteristics, laboratory measurements, and death.

## Conclusion

In conclusion, we showed that cardiovascular disease, prior catheter use, levels of albumin, hsCRP, and fetuin-A were associated with primary patency loss in both patients with a fistula and a graft. Current guidelines for prevention of vascular access failure recommend uniform surveillance of all patients [31]. The results of our study might lead to a more directed approach for surveillance techniques. The observed risk factors for primary patency loss could be used to focus on specific patient groups for more intensive surveillance. Furthermore, we showed that graft use as compared with fistula use was associated with an increased risk of primary patency and an increased mortality risk.

## Competing interest

All authors declared that they have no competing interest.

## Authors’ contributions

GO, JIR and MV formed the study concept, analyzed the data, interpreted the results, and drafted the manuscript. CYV and FRR interpreted the results and revised the manuscript. FWD, RTK, and EWB formed the study concept, collected the data and revised the manuscript. All authors read and approved the final manuscript.

## Pre-publication history

The pre-publication history for this paper can be accessed here:

http://www.biomedcentral.com/1471-2369/14/79/prepub
